# Multiple calmodulin genes of the Pacific abalone, *Haliotis discus hannai* (Mollusca: Vetigastropoda: Haliotidae)

**DOI:** 10.1080/19768354.2018.1509126

**Published:** 2018-09-19

**Authors:** Han Kyu Lim, Jong Kyu Lee, Gun-Do Kim, Tae Hyug Jeong

**Affiliations:** a Department of Marine and Fisheries Resources, Mokpo National University, Korea; b Department of Microbiology, Pukyong National University, Korea

**Keywords:** Calmodulin, calcium signaling, expression profiling, gene structure, invertebrate

## Abstract

In this study, we identified four canonical calmodulin genes in the Pacific abalone, *Haliotis discus hannai*. Their full-length cDNAs were variable in the 5′ and 3′ untranslated regions, but highly similar (91–97%) in the coding region. Each of the genes encoded 149 amino acids, with 93–97% similarity among themselves and 94–98% similarity with human CAM I. There were 54 substitutions distributed unevenly throughout the coding regions, found mostly in the third codon position. Gene structure analysis revealed that each of the calmodulin genes comprised five exons and four introns. The intron positions and phases were identical and there were no introns in the fourth exon. The corresponding introns differed in their sequences and sizes. Expression profiles of nine tissues from abalone revealed that the calmodulin genes were transcribed in common in gill and mantle tissue, but differentially in the other tissues. A phylogenetic analysis based on the amino acid sequences revealed that calmodulin C was the most common isoform in Gastropoda and calmodulin was the most diverged isoform. An *in silico* analysis of the calmodulin genes identified paralogous genes in other *Haliotis* species, indicating that gene duplication might have occurred in the last common ancestor of *Haliotis*.

**Abbreviations:** ORF: open reading frame; RACE: random amplification of cDNA end; TSA: transcriptome shotgun assembly; UTR: untranslated region

## Introduction

Abalones of the genus *Haliotis* are major marine invertebrates in the fisheries and aquaculture industries worldwide. The Pacific abalone, *Haliotis discus hannai*, is an especially important species in Asia. To increase its commercial productivity, research has been conducted on its growth (Choi et al. 2015), immunity (Bathige et al. 2014), and biomineralization (Shen et al. 1997). Quantitative traits related to growth of the soft body and shell of abalone are among the most important characteristics for successful aquaculture and breeding programs.

As a secondary messenger, calcium mediates a variety of calcium signaling pathways related to growth, development, proliferation, the cell cycle, and movement (Weinman et al. 1991; Takuwa et al. 1995; Chin & Means 2000). Calcium ions are also the primary inorganic constituents of the shell (Shen et al. 1997). Calcium-binding proteins (CBPs) participate in these processes by binding to calcium ions. The CBPs of the EF-hand superfamily, which have 2–12 Ca^2+^-binding EF-hand motifs, alter many target proteins involved in the signal transduction pathway in response to changes in free calcium ion levels. The prototypical EF-hand protein calmodulin (CaM) transduces the calcium signal to target proteins downstream, changing their activity in a calcium-dependent manner.

CaM is found in all eukaryotic cells and its sequence is highly conserved (Toutenhoofd & Strehler 2000). The number of canonical *CaM* genes varies among phyla. There are three bona fide *CaM* genes in mammals (Fischer et al. 1988; Nojima 1989; Skinner et al. 1994), while there are two in chicken (Putkey et al. 1983; Ye & Berchtold 1997), three in turtle (Shimoda et al. 2002), four in medaka (Matsuo et al. 1992), and six in zebrafish (Friedberg & Taliaferro 2005), all of which have the same amino acid sequence. Plants such as *Arabidopsis* and rice have several CaM isoforms (Yamakawa et al. 2001; McCormack & Braam 2003; Boonburapong & Buaboocha 2007; Zhao et al. 2013). Only one *CaM* gene has been reported in most invertebrates (Doyle et al. 1990; Swanson et al. 1990; Di Gregorio et al. 1998; Yuasa et al. 2001; Gao et al. 2009; Ji et al. 2011; Zeng et al. 2012; Chen, Wang, Matsumura, et al. 2012; Chen, Wang, Qian 2012; Li et al. 2014), whereas sea urchin, sea squirt, amphioxus, and flatworm each have two *CaM* genes (Hardy et al. 1988; Yuasa et al. 1999; Karabinos & Bhattacharya 2000; Taft & Yoshino 2011). Even using sensitive detection methods, no additional *CaM* genes have been found in fruit fly (Doyle et al. 1990). Russell et al. (2007) reported that liver fluke has two CaM-like proteins, one of which is a canonical CaM with high similarity (98%) to human CAM, while the other is CaM-like with lower similarity (41%). Systems consisting of multiple *CaM* genes seem to be limited to plants and the Deuterostomia lineage of animals; however, intron analyses have suggested that *Littorina* spp. and *Mytilus* spp. may have multiple *CaM* genes (Corte-Real et al. 1994; Simpson et al. 2005). Few studies have been conducted on *CaM* in Gastropoda aside from the sea hare (Swanson et al. 1990).

In addition to the diverse proteins related to growth of the soft body and matrix proteins involved in formation of the organic parts of the shell in abalone, understanding the regulatory, functional, and genetic properties of CaM and CaM-like proteins in calcium metabolism is an attractive research topic. CaM and CaM-like protein were reported to be involved in the shell formation of *Pinctada fucata* (Li et al. 2004; Li et al. 2005; Huang et al. 2007).

Here, we describe the presence and structural organization of four non-allelic *CaM* genes in Pacific abalone, representing the first finding of a multigene system for CaM in invertebrates.

## Materials and methods

### In silico identification of putative cam genes in haliotis

We obtained 24,746 expressed sequence tags (ESTs) and 33,153 nucleotide sequences of the genus *Haliotis* (*H. tuberculata, H. asinina, H. diversicolor, H. midae, H. discus, H. rufescens*) from the National Center for Biotechnology Information (NCBI) EST and nucleotide databases (https://www.ncbi.nlm.nih.gov/). A total of 117,537 reference contigs of *H. discus hannai* were also included in this analysis (Choi et al. 2015). A local nucleotide database including a total of 175,453 sequences was constructed and used as a primary screen for putative *CaM* genes via a tblastn search with default search parameters using the amino acid sequence of human CAM I (GenBank Accession No. NP_008819) as a query. Subsequently, to distinguish other non-canonical EF-hand proteins or calcium-binding proteins from canonical CaM, we selected sequences encoding proteins that had >90% similarity with human CAM I (McCormack & Braam 2003). After manually searching for open reading frames (ORFs) and untranslated regions (UTRs), we discarded sequences without both 5′- and 3′-UTRs. Then a multiple nucleotide alignment of the final selected candidates was performed with the ClustalW program and misaligned regions were corrected manually due to variations in sequence length. The local database construction, tblastn search, and alignment were performed using functions implemented in BioEdit software (http://www.mbio.ncsu.edu/BioEdit/bioedit.html).

### RT-PCR analysis for tissue-specific expression profiles

Tissue-specific expression profiles were determined for ganglion, tentacle, gill, heart, hepatopancreas, digestive tract, gonad, mantle, and muscle tissues dissected from an adult *H. discus hannai*. The abalone was reared in an aquaculture tank at the Genetics and Breeding Research Center of the National Fisheries Research and Development Institute (NFRDI, Geoje, Korea) and was removed and then dissected immediately. The dissected tissues were immersed in RNAlater (Sigma, St. Louis, MO, USA) solution and transported to the laboratory. Total RNA was extracted from each tissue using TRI Reagent (Sigma, St. Louis, MO, USA) followed by the Hybrid-R kit (GeneAll, Seoul, Korea). Then, the first strand of complementary DNA (cDNA) was synthesized using the M-MLV cDNA Synthesis Kit (Enzynomics, Daejeon, Korea) following the manufacturer’s protocol. The cDNAs were normalized to ribosomal protein L3 and used as templates for reverse transcriptase (RT)-PCR. Based on multiple alignments, specific forward and reverse primers for each group were designed based on the 5′- and 3′-UTRs, respectively (Table 1). For the forward primer of *CaM D* without the 5′ end, CAM-F primer from the conserved region of *CaM A*, *CaM B*, and *CaM C* was used. RT-PCR was conducted in HiQ-PCR Mix (GenoTech Corp., Daejeon, Korea) using the following conditions: initial denaturation at 95°C for 3 min, 25 cycles of denaturation at 95°C for 30 s, annealing for 30 s at the optimum temperature of each primer pair, extension at 72°C for 30 s, and a final extension at 72°C for 5 min. As an internal positive control, ribosomal protein L3 was amplified over 25 cycles consisting of denaturation at 95°C for 30 s, annealing at 55°C for 30 s, and extension at 72°C for 30 s.

**Table 1.** Primers used in this study.

	Primer	sequence (5′ to 3′)	
CaM A	CaMA-F	TGGACGTCGTGTTCAGTAAAG	RT-PCR, genomic PCR, 3' RACE
	CaMA-R	GACAGACGAGGATGACAGAAAG	RT-PCR, genomic PCR
			
CaM B	CaMB-F	CGAGTCACAGACGACAGAAA	RT-PCR, 5′ genomic fragment, 3′-RACE
	CaMB-R	CATGACGTCTTTGCTGTCAATC	RT-PCR, 3′ genomic fragment
	CaMB-e2F	CAGACCAGCTGACAGAGGAACAGA	3′ genomic fragment
	CaMB-e2R	CAGCGATCTGTTCCTCTGTCAGCT	5′ genomic fragment
			
CaM C	CaMC-F	CAGTGTCGGAAGAACGGATAG	RT-PCR, 5′ genomic fragment, 3′-RACE
	CaMC-R	GCAGATAGCCCTGTACTCAAC	RT-PCR, genomic PCR
	CaMC-e2F	CGGACCAGTTGACTGAAGAGCAGA	3′ genomic fragment
	CaMC-e2R	CTGCAATCTGCTCTTCAGTCAACTGG	5′ genomic fragment
	CaMC-i4R	CAACAAATGGGAATGACATAAATGGC	3′ genomic fragment
			
CaM D	CaMD-F1	GTCGACGGTGGTGTCACTCT	Downstream of exon 1
	CaMD-F2	ACGTCGCTGACAACACTTATAG	RT-PCR, downstream of exon 1, 3′'-RACE
	CaMD-R	AAAGCTGCCCCAACATAAGATGGTT	RT-PCR, genomic PCR, 5′-RACE
	CaMD-e2F	GCAACCGATCTGACGGAAGAG	3′ genomic fragment
	CaMD-i2R1	ATGGGCCATGGGTGAAACAT	Upstream of intron 2
	CaMD-i2R2	AAGGACGACATTCTGAGCCG	Upstream of intron 2
			
Common	CaM-F	CAAGACATGATCAACGAAGT	RT-PCR, genomic PCR, 3′-RACE

### Cloning the full-length cDNA of CaM genes with RACE PCR

Because *CaM A*, *CaM B*, and *CaM C* had 5′-UTRs, their 3′ ends were identified with 3′-random amplification of cDNA ends (RACE) using the SMARTer RACE 5′/3′ Kit (Clontech Laboratories, Mountain View, CA, USA) following the manufacturer’s protocol. The specific forward primers of each *CaM* were applied to the GSP1 primer in 3′-RACE. Due to the absence of a 5′ end for *CaM D*, 5′-RACE was applied first with a specific reverse primer using the SMARTer kit, and then 3′-RACE was created using a specific forward primer designed from the 5′-UTR obtained from the 5′-RACE. Total RNA isolated from adult abalone gill tissue was used as the template. Each gel-eluted PCR product was cloned into T-vector and sequenced.

### Amplification and sequencing of CaM genomic fragments

To confirm the exon/intron structure, we amplified genomic fragments of each *CaM* gene using long PCR. First, genomic DNA was extracted from mantle tissue using the PrimePrep Genomic DNA Kit (GeNet BIO, Korea) following the manufacturer’s protocol. During the first trial, we attempted to amplify 3′ half fragments using pairs of common CAD-F primers and each specific reverse primer using LA Taq (TAKARA, Japan). The PCR conditions were optimized to amplify products < 12 kb long. The amplified products of *CaM A*, *CaM B*, *CaM C*, and *CaM D* were ∼1.7, 3.4, 1.6, and 3.5 kb long, respectively. During the second trial, we attempted to amplify whole fragments using each specific forward primer of the 5′-UTR and each of the same specific reverse primers used during first trial. From this trial, we only obtained a ∼7-kb fragment of *CaM A*. Because the *CaM B*, *CaM C*, and *CaM D* 3′ fragments obtained during the first trial contained exon 3 and downstream sequences, we speculated that intron 1 or intron 2 of *CaM B*, *CaM C*, and *CaM D* were large. Therefore, during the third trial to amplify the remainder of *CaM B*, *CaM C*, and *CaM D*, we designed the forward primers to target the exon 2 region and conducted amplification using the same reverse primers for each gene. We obtained fragments of ∼10 kb for *CaM B* and ∼4 kb for *CaM D*, but did not obtain any PCR product for *CaM C*. During the fourth trial, we amplified 5′ fragments of *CaM B*, *CaM C*, and *CaM D* encompassing exon 1 to exon 2 using the specific forward primers used during the second trial and the reverse primers used during the third trial. From this, we obtained fragments of ∼10 kb for *CaM B* and ∼2.1 kb for *CaM C*. During this trial, the whole fragment of *CaM A* was cloned and fully sequenced. The 5′ and 3′ fragments of *CaM B* were cloned and partially sequenced at the exon/intron boundary regions. During the fifth trial for *CaM C*, the remaining intron 3 region of *CaM C* was amplified using the specific forward primer of the exon 2 region and the reverse primers for intron 4. The resulting product size was ∼11 kb. The 2.1-kb fragment of the 5′ and 1.6-kb fragment of the 3′ ends were cloned and sequenced. The 11-kb fragment was cloned and partially sequenced at the exon/intron boundary regions. The remaining exon 1 and intron 1 region of *CaM D* was not amplified in any additional trials using long PCR. The 4-kb fragment of *CaM D* was cloned and sequenced during the third trial. We used the restriction cutting independent method (Rudi et al. 1999) with some modifications to identify intron 1 of *CaM D*. Briefly, CaMD-F1 primer, targeting downstream of exon 1, and CaMD-i2R1 primer, targeting upstream of exon 2, were used in a linear amplification reaction. The purified single-strand amplification products were tailed by terminal deoxynucleotidyl transferase (TAKARA, Japan). The poly-guanine primer (5′-GGCCACGCGTCGACTAGTACGGGGGGGGGGGGGGGG) and both CaMD-F2 and CaMD-i2R2 were used for the amplification. The products were cloned into T-vector and several clones were sequenced. We confirmed the sequence for *CaM D* by comparing the 5′-UTR, exon 2, and intron 2 sequences of *CaM D*.

### Phylogenetic analyses

To examine the phylogenetic relationships, we obtained *CaM* genes (Table 2) from various species belonging to Gastropoda, Cephalopoda, Lingulata, and Polychaeta from the EST, Transcriptome Shotgun Assembly (TSA), and draft genome databases of NCBI (https://www.ncbi.nlm.nih.gov/). For five Heterobranchia species and two Caenogastropoda species, two *CaM* isoform genes were included, which were mostly TSAs. In *Biomphalaria glabrata* (Heterobranchia), one isoform (GenBank Accession No. XM_013208033) was a predicted gene from genome scaffold 868, which had several corresponding EST sequences, while the other isoform (GenBank Accession No. XM_013211563) was predicted from genome scaffold 161 with a longer C-terminus, which might have derived from a misprediction of exon 5 due to missing sequences in the draft genome compared to its corresponding ESTs; therefore, we used one EST sequence (GenBank Accession No. FC859952). The deduced sequences of these *CaM* genes were all 149 amino acids in length, so there was no need for alignment. For downstream analyses, MEGA7 software was used (http://www.megasoftware.net/). The best amino acid substitution model among 56 candidate models was the LG + G model, which had the lowest BIC scores (1929.3) and best described the substitution pattern. A phylogenetic tree was constructed using the maximum likelihood (ML) method. Bootstrap values were estimated using 1,000 replicates.

**Table 2.** Sequences of calmodulin genes used in phylogenetic reconstruction.

Phylum	Class		Species		GenBank acc. no.
Streptophyta			*Arabidopsis thaliana*	1	NM_123137
				2	M38380
				6	NM_180529
				7	NM_114249
			*Nicotiana tabacum*	1	AB050837
				3	AB050839
				9	AB050845
Annelida			*Hydroides elegans*		JN688261
Brachiopoda			*Lingula anatina*		XM_013526062
Mollusca	Cephalopoda		*Octopus bimaculoides*		XM_014931506
	Gastropoda	Patellogastropoda	*Lottia gigantea*		XM_009057815
		Vetigastropoda	*Haliotis discus hannai*	A	KU991738
				B	KU991739
				C	KU991740
				D	KU991741
		Heterobranchia	*Aplysia californica*		NM_001204580
			*Biomphalaria glabrata*	1	XM_013208033
				2	FC859952
			*Lymnaea stagnalis*	1	FX183278
				2	FX191330
			*Plakobranchus ocellatus*	1	HP167232
				2	HP200164
			*Elysia timida*	1	GBRM01065559
				2	GBRM01035007
			*Elysia cornigera*	1	GBRW01069283
				2	GBRW01140909
		Caenogastropoda	*Cipangopaludina cathayensis*	1	GCEL01069670
				2	GCEL01082765
			*Pomacea canaliculata*	1	GBZZ01001913
				2	GBZZ01064483

The numbers of calmodulin isoforms of Gastropoda is arbitrary.

## Results

### Isolation and characterization of four cDNAs encoding abalone cams

Primary screening for putative *CaM* genes and *CaM*-like genes via tblast search resulted in a variety of EST and TSA sequences encoding proteins with >50% similarity to human CAM I, as most EF-hand proteins have two or more EF-hand motifs. To discriminate true CaM from other EF-hand proteins in *Arabidopsis* (McCormack & Braam 2003), we selected sequences encoding amino acids with >90% similarity. After discarding sequences without both 5′- and 3′-UTRs, we obtained about 20 sequences. Next, these 20 nucleotide sequences were aligned. The sequences clustered into four distinct groups (A, B, C, and D) based on dissimilarities in their UTR regions.

**Figure 1.**
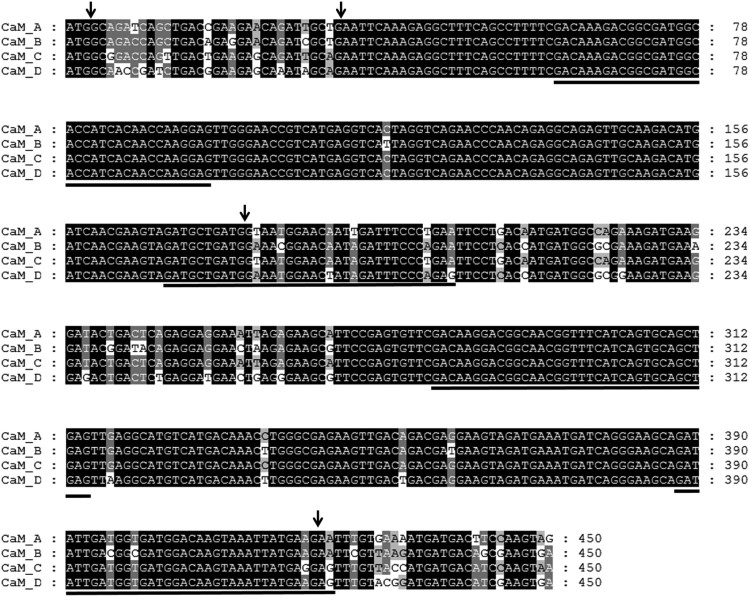
Comparison of the nucleotide sequences of abalone *CaM* cDNAs in the coding region. Identical nucleotides are darkly shaded. Nucleotide numbers are shown on the right. Downward arrows indicate intron positions. The four EF-hand Ca^2+^-binding loops are underlined.

**Figure 2.**
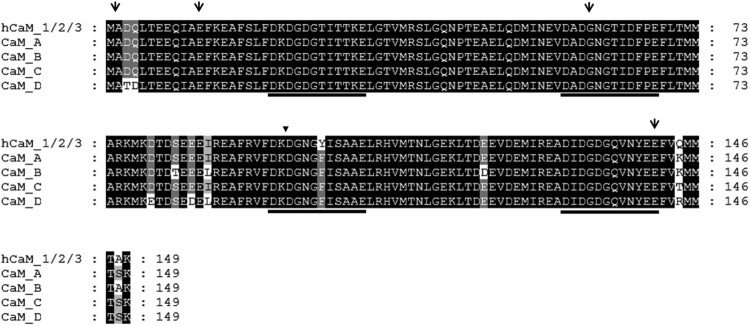
Comparison of the amino acid sequences of abalone CaMs with human CaM 1/2/3*.* Identical amino acid residues are darkly shaded, similar amino acids are lightly shaded, and unrelated residues have a white background. Amino acid numbers are shown on the right. Downward arrows indicate intron positions. One inverted triangle indicates the intron position found only in human CAMs. The four EF-hand Ca^2+^-binding loops are underlined. Human CAM 1/2/3 are represented (GenBank accession nos. NP_008819, NP_001743, and NP_005175, respectively).

Using 5′- and 3′-RACE PCR with primer pairs based on the 5′- and 3′-UTRs of each group, we obtained four different full-length *CaM* cDNAs, designated as *CaM A*, *CaM B*, *CaM C*, and *CaM D*, according to the derived group, which are deposited in GenBank (*CaM A,* GenBank Accession No. KU991738; *CaM B,* KU991739; *CaM C,* KU991740; and *CaM D,* KU991741). Their nucleotide characteristics are summarized in Table 3. A nucleotide comparison revealed that their coding regions were 91–97% similar, whereas there were no significant sequence similarities in the 5′- and 3′-UTRs. There were 54 base substitutions in the coding region, which were distributed unevenly and found mostly in exon 2 (13/54), the 5′-half of exon 4 (22/54), and exon 5 (11/54), with only one found in exon 3 (Figure 1). The deduced amino acid sequences of the abalone *CaM* genes exhibited 93–97% similarity among themselves and 94–98% similarity with human CAM I (Figure 2). There were three (CaM A/B), one (CaM A/C), and seven (CaM A/D) variable residues. There were no different residues in the four EF-hand loops. The variations between the third (T) and fourth (D) residues of CaM D have not been reported in other CaM proteins to date.

**Table 3.** Summary of the characteristics of the four *CaM* cDNAs of Pacific abalone.

	5′-UTR	ORF	Stop codon	3′-UTR	polyA signal	Total
CaM A	89	447	TGA	384	ATTAAA	943
CaM B	76	447	TGA	396	AATAAA	942
CaM C	64	447	TAA	1517	CATAAA	2051
CaM D	82	447	TGA	165	GATAAA	717

### Expression profiling

**Figure 3.**
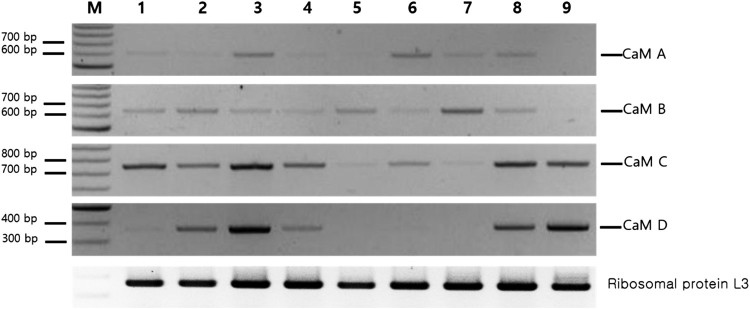
Expression profiles of abalone *CaM* genes from nine tissues assessed by semi-quantitative reverse transcriptase PCR (RT-PCR) analysis. Expression of ribosomal protein L3 was used for normalization. M, 100-bp size marker; 1, ganglion; 2, tentacle; 3, gill; 4, heart; 5, hepatopancreas; 6, digestive tract; 7, gonad; 8, mantle; 9, muscle.

To examine tissue-specific expression of the four *CaM* genes, we performed semi-quantitative RT-PCR analyses using nine tissues from an individual abalone (Figure 3). Ribosomal protein L3 was used as an internal control (Choi et al. 2015). *CaM A* was expressed at relatively high levels in the gill and digestive gland, and was expressed in all other tissues except the hepatopancreas and muscle. *CaM B* was ubiquitous in all tissues examined, but was higher in the gonad and absent from muscle. *CaM C* was ubiquitous in all tissues, but was relatively low in the hepatopancreas and gonad. *CaM D* was transcribed in the tentacle, heart, and mantle, with higher levels in gill and muscle, and its transcripts were rare in the ganglion, hepatopancreas, digestive track, and gonad. All four *CaM* genes were expressed in the gill and mantle, which play roles in primary calcium uptake and secretion during shell formation, respectively (Rousseau et al. 2003; Li et al. 2004). While *CaM C* and *CaM D* expression levels were relatively high in muscle tissue, *CaM A* and *CaM B* were not expressed in muscle. In the hepatopancreas, *CaM B* was the major transcript, although there were also low levels of *CaM C* expression. *CaM D* exhibited relatively low expression in gill, muscle, tentacle, and heart tissues.

### Organization of the calmodulin genes

**Figure 4.**
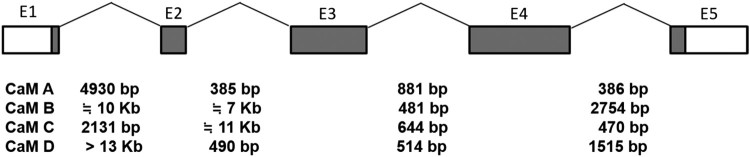
Gene structures of the abalone *CaM* genes. Exons are shown as boxes in which the coding regions are shaded and untranslated regions are white. Each exon is represented, including E1 , E2 , E3 , E4 , and E5. Intron lengths of each gene are shown at the bottom.

To examine the gene structure, we used long PCR to amplify the genomic fragments containing each *CaM* gene. Genomic fragments of *CaM A*, *CaM B*, and *CaM C* were successfully amplified containing partial 5′- and 3′-UTRs with introns 1, 2, 3, and 4, and *CaM D* containing exon 2 and a partial 3′-UTR. Because the fragment containing exon 1 and intron 1 for *CaM D* was not amplified with long PCR due to its longer length of >13 kb, we obtained a partial 5′ splice junction region (901 bp) and 3′ splice junction region (341 bp). The long introns (intron 1 and 2 of *CaM B* and intron 2 of *CaM C)* were not fully sequenced and their lengths were approximated with agarose gel electrophoresis of the PCR products. These four genome sequences are deposited in GenBank (*CaM A*, GenBank Accession No. KU991742; *CaM B*, KU991743; *CaM C*, KU991744; and *CaM D*, KU991745). No substitutions were observed between the cDNAs and genomic sequences of each *CaM* gene. The gene structures are shown in Figure 4. All introns showed the canonical GT/AG rule in the 5′ and 3′ exon/intron boundaries. The exon/intron structures of all abalone *CaM* genes were the same as those of the sea squirt (Yuasa et al. 1999), whereas the corresponding intron sequences and lengths among four *CaM* genes were variable. The first exon of the four *CaM* genes encoded only the first start codon.

### Phylogenetic reconstruction

**Figure 5.**
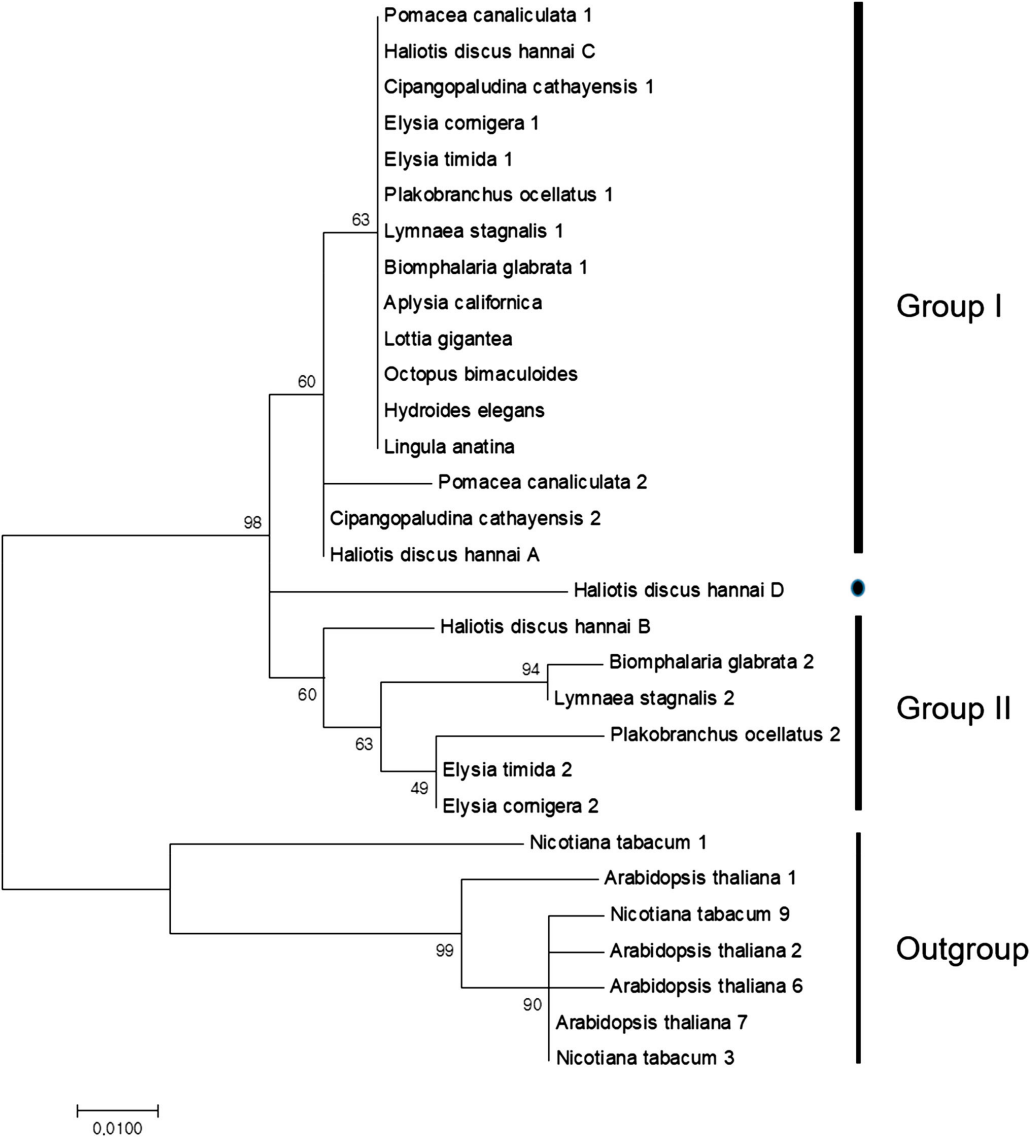
Phylogenetic relationship of CaM proteins. We used 30 amino acids to construct a phylogenetic tree using the maximum likelihood (ML) method with the LG + G model in MEGA7. The bootstrap percentages from 1000 bootstrap replications are shown next to the branches. The sequence names in the tree are shown in Table 2.

We performed phylogenetic analyses using the amino acids of the four abalone CaM proteins and 26 CaM proteins from other species. In the final tree (Figure 5), the two isoforms of CaM were included for such species as *B. glabrata* of Heterobranchia and *Cipangopaludina cathayensis* of Caenogastropoda. The plant CaM isoforms of *Arabidopsis* and common tobacco were used as an outgroup. There were two main groups (Groups I and II). Group I consisted of 13 CaM proteins including CaM A and 12 other CaMs from all species considered, such as CaM C, *C. cathayensis* 2 and *Pomacea canaliculata* 2. Group II included CaM B and one of the two CaM proteins of Heterobranchia. CaM D was not grouped and was separate from the other CaM proteins. CaM A and 12 CaMs in Group I with identical amino acid sequences appeared to be ancient and ordinary due to their existence in all species examined. The second CaM proteins of Heterobranchia and Caenogastropoda were not clustered together. We assumed that the members of Groups I and II had derived from a gene duplication event and diverged differentially into each lineage. We assumed CaM D to be the most diversified protein and specific to the *Haliotis* lineage.

## Discussion

In vertebrates including humans, several *bona fide CaM* genes are present, located in different chromosomes. In invertebrates, however, this multigene CaM is not common, and is known in only a few cases.

We obtained numerous sequences encoding EF-hand proteins from a primary tblastn search. Using the strict criteria applied to *Arabidopsis* to discriminate true CaM from other EF-hand proteins (McCormack & Braam 2003), we identified four groups of sequences for true CaM. Based on these partial sequences, we obtained four full-length cDNAs using RACE PCR, encoding 149 amino acids with 4 EF-hand motifs. Although the 5′- and 3′-UTR sequences were distinct between them, we might not exclude that they are alternative splicing forms of a certain gene because the substitutions were distributed unevenly and highly conserved in exon 3. Then, we analyzed their gene structures. These analyses showed that all of the genes had the identical gene structure of exons and introns in position and phase but differ in intron sequences, indicating that they are not allelic or splicing variants of one gene. In addition, they were transcribed differentially in different tissues. Taken together, we concluded that these four genes were canonical *CaM* genes. To the best of our knowledge, this is the first report to identify and characterize four *CaM* genes in *Haliotis* (Vetigastropoda). It remains unknown whether these four *CaM* genes are located on separate chromosomes or as tandem repeats on the same chromosome

Although they were not full-length in most instances, transcripts corresponding to the four *CaM* genes of *H. discus hannai* were identified in ESTs or TSAs of other *Haliotis* species. These transcripts had highly similar identities in the 5′- or 3′-UTRs, including orthologs of *CaM A*, *CaM B*, and *CaM C* in *H. rufescens*, *H. midae*, *H. diversicolor*, *H. tuberculata*, and *H. asinina* and an ortholog of *CaM D* in *H. rufescens*, which suggests that gene duplication of *CaM* might have occurred in the last common ancestor of these *Haliotis* species. Amino acid-based phylogenetic analyses revealed that CaM C is likely the most primitive version, as it was the most common type that we examined, while CaM D was the most divergent. A multigene system requiring at least two isoforms might exist in Gastropoda. In the gastropod database, we found two putative isoforms in five Heterobranchia species and two Caenogastropoda species, among which each isoform differed in their 5′- or 3′-UTR sequences. In *B. glabrata*, the genomic sequences of two isoforms were also available.

The major difference compared to vertebrates is that abalone *CaM* genes do not encode identical proteins. Vertebrates exhibit a special case of genetic redundancy in their temporal and spatial expression of *CaM*, whereby three *CaM* genes encode identical proteins (Toutenhoofd & Strehler 2000; Friedberg & Rhoads 2001). Therefore, we speculate that abalone *CaM* genes may have experienced similar evolutionary pressures as vertebrates to maintain the four paralogs. However, their functional categories are more similar to the multigene family *CaM* members of higher plants, which encode several isoforms with spatially and temporally variable transcription and which respond to many different stimuli (Ranty et al. 2006). We assumed that because the development and growth of the edible soft body of the abalone is limited to the size of the shell for protection from predators, the growth of the shell is also important. The abalone shell consists primarily of inorganic composites (calcium carbonates) with some organic molecules. Many studies have investigated the mechanism of shell formation, which have identified numerous proteins including matrix proteins. On the other hand, the calcium used for building the exoskeleton is not sourced directly from seawater but rather is taken up, transported, and excreted at sites of shell formation. Further studies on the temporal expression patterns during abalone development, as well as physiological and biochemical characterizations, would help to elucidate the divergent roles of the four abalone *CaM* genes in regulatory mechanisms related to growth, calcium metabolism, and biomineralization.

In past decades, studies focused on cellular function, structure, and signaling in vertebrates, while the identification of the *CaM* gene in invertebrates was restricted to early studies using traditional methods to identify one *CaM* gene in invertebrates. To identify one or more true *CaM* genes in individual species, thorough studies using massive sequences derived from methods such as transcriptomic and genomic sequencing are necessary. Meanwhile, although we used transcriptomic data from short reads, we did not obtain the corresponding full-length contigs of four abalone *CaM* genes directly through *de novo* assembly, even though they encode relatively short amino acid sequences. The contig for *CaM A* was not identified completely from the reference contigs; the contigs for *CaM B* and *CaM D* were fragmented and some portions were missing; and the contig for *CaM C* contained ambiguous sequences in the middle. The high nucleotide identities in the coding regions of each abalone *CaM* gene seem to make distinct contigs from the homogeneous short reads when *de novo* assembled. Even if a method to detect transcript variants was used, distinguishing among highly homologous isoforms, such as the abalone *CaM* genes, would be challenging. In addition to identifying alternative isoforms of a certain gene, this sequencing method can be used for long reads. In addition, it would be difficult to predict the *CaM* gene properly in genome sequences if there is no evidence of the transcript, because the first exon only encodes the start codon. If several copies of *CaM* genes exist in a species, it is insufficient to only annotate *CaM* or *CaM*-like genes in a draft genome or transcriptomes using automatic prediction software. Therefore, additional comprehensive evidence is needed to confirm that either one *CaM* gene or a multigene family exists in other invertebrates.

In summary, we identified and characterized four *bona fide CaM* genes from the Pacific abalone by cloning cDNAs and genomic fragments, and conducting expression profiling. Compared to vertebrate *CaM* genes, abalone *CaM* genes do not encode the same amino acids, and are more similar to the multigene system of higher plants. Among the four isoforms, *CaM C* might be the ordinary sequence from which the others diverged after gene duplication. At least three *CaM* transcripts could be found in other *Haliotis* species, indicating that gene duplication might have occurred before the divergence of *Haliotis* species.
